# Efficacy of a topical application of Certifect^®^ (fipronil 6.26% w/v, amitraz 7.48% w/v, (S)-methoprene 5.63% w/v) for the treatment of canine generalized demodicosis

**DOI:** 10.1051/parasite/2013046

**Published:** 2013-11-21

**Authors:** Josephus Fourie, Pascal Dumont, Lénaïg Halos, Frederic Beugnet, Matthias Pollmeier

**Affiliations:** 1 Merial S.A.S. 29 avenue Tony Garnier 69630 Lyon France; 2 Clinvet International (Pty) Ltd PO Box 11186 9321 Universitas, South Africa

**Keywords:** *Demodex canis*, demodicosis, treatment, fipronil/amitraz/(S)-methoprene, dog

## Abstract

The efficacy of the treatment with Certifect ® (containing fipronil 6.26% w/v, amitraz 7.48% w/v, (S)-methoprene 5.63% w/v) applied topically was assessed in 18 dogs diagnosed with clinical generalized demodicosis. Three treatment regimens were compared over a 3-month period. Starting at Day 0, dogs were treated monthly (group 1) or every two weeks (group 2) with the combination of fipronil, amitraz, and (S)-methoprene or with monthly topical applications of the combination of amitraz and metaflumizone (group 3, reference treatment). Clinical examinations including deep skin scrapings were performed every month in order to evaluate the resolution of clinical signs and the reduction in mite counts. On Day 84, the percentage reduction of mite counts in group 1 was 99.8%, whereas no *Demodex canis* could be detected in groups 2 and 3 (i.e. 100% parasitological efficacy). As a result of the *Demodex* mite count reduction, the skin condition of the dogs improved significantly in all groups. This study illustrates, that both monthly and bi-weekly treatments with Certifect were effective in treating dogs with generalized demodicosis over a 3-month period.

## Abstract (fr)

L’efficacité du traitement topique avec Certifect® (combinaison de fipronil 6,26 % w/v, amitraz 7,48 % w/v, et (S)-methoprene 5,63 % w/v) a été évaluée chez 18 chiens atteints de démodécie généralisée et porteurs de *Demodex canis*. Trois protocoles de traitement ont été comparés sur une période de 3 mois. À partir du jour 0, les chiens du groupe 1 ont été traités 3 fois à un mois d’intervalle avec la combinaison d’amitraz, de fipronil et de (S)-méthoprène; les chiens du groupe 2 ont été traités avec la même combinaison administrée à des intervalles de 2 semaines et les chiens du groupe 3 ont été traités par application topique mensuelle d’une combinaison commerciale d’amitraz et de métaflumizone ayant l’indication pour le traitement de la démodécie canine. L’efficacité des différents protocoles a été évaluée au cours d’examens cliniques mensuels accompagnés de raclages cutanés profonds. Au jour 84, le pourcentage de réduction parasitaire dans le groupe 1 était de 99.8 %, il était de 100 % dans les groupes 2 et 3. Les lésions dermatologiques étaient significativement réduites dans les 3 groupes. Cette étude confirme que l’application topique de Certifect pendant 3 mois à un intervalle de 2 semaines ou 1 mois permet de traiter avec succès les chiens atteints de démodécie généralisée.

## Introduction

Demodicosis is a parasitic inflammatory skin disease of dogs caused by an excessive proliferation of *Demodex canis* Leydig, 1859 [8]. A small number of mites may constitute a normal component of the dog’s skin fauna [15,16], but a proliferation of mites can lead to serious disease. The parasite is not considered contagious except during a few days after birth, when puppies acquire mites through direct skin contact from their mother. Three morphologically different types of *Demodex* have been described and named as species by some authors (*D. canis*, *D. injai*, and *D. cornei*) but a final consensus on taxonomy will require molecular testing [5,9,15]. Published data indicate similar efficacy of reported treatments regardless of the *Demodex* type [9].

Canine demodicosis can be divided into two clinical manifestations. The localized form appears as small patches of alopecia and mild erythema in young dogs. It generally regresses spontaneously without treatment. The generalized form of demodicosis is more severe and can even be fatal. It may develop from the localized condition or occur in older animals, especially those undergoing severe stress or with underlying diseases. The definition of localized versus generalized demodicosis has been a matter of debate. A recent committee of experts considered demodicosis as localized if there are no more than four lesions with a diameter of up to 2.5 cm in width [5, 9]. Canine generalized demodicosis is frequently seen in practice [13] and is characterized by five or more affected areas or by lesions covering an entire region of the body, and/or pododemodicosis involving two or more paws [3]. The affected areas are erythematous, with comedones, hair loss, follicular papules to pustules and scales. Lymphadenopathy is commonly associated with the disease and secondary bacterial infections are very frequent [5]. Although some young dogs with an early generalized form can self-cure naturally, it is impossible to clinically ascertain which animals will progress to the more severe state. The diagnosis is typically based on clinical signs and is confirmed by the presence of mites in deep skin scrapings. Although *Demodex* mites are part of the normal microfauna, it is uncommon to find the mites, even by performing several deep skin scrapings. If a mite is found, this should raise suspicion and additional skin scrapings should be performed. Finding more than one mite is strongly suggestive of clinical demodicosis [5, 9].

Despite recent advances with new acaricidal drugs, generalized demodicosis remains a very challenging disease to treat effectively. Amitraz, as a rinse or sponge -on, has been approved for the treatment of canine generalized demodicosis in many countries for decades. Several amitraz-based protocols have been described at various concentrations and frequencies. Efficacy data are reported to be variable, it is time-consuming, and there may be safety issues [11]. Protocols based on daily to weekly oral or subcutaneous injections of macrocyclic lactones including ivermectin, doramectin, and moxidectin have been published but represent off-label use with potential for toxicity, especially in dogs mutated for MDR-1 (P-glycoprotein deficiency), especially including colley breeds [9, 11]. Daily oral milbemycin oxime at a dose of 500 mg/kg is registered in many countries for the treatment of canine demodicosis [7].

The most recent registered treatments for demodicosis include topical products that help improve owner compliance, and therefore increase the rate of success. These drugs, applied as a spot-on at monthly or bi-weekly intervals, contain well-known active compounds like amitraz (combined with the insecticide metaflumizone) or moxidectin (combined with the insecticide imidacloprid) [2, 3, 6, 10]. These new alternatives provide a safer and more convenient approach to cure this disease. Whatever the choice of the antiparasitic drug, the duration of the treatment of demodicosis usually requires 3 months or more.

Certifect^®^ is a spot-on formulation that combines fipronil, amitraz, and (S)-methoprene. The addition of amitraz to fipronil has been shown to significantly potentiate the acaricidal effects of fipronil [12, 14]. The product has been recently demonstrated to be active against sarcoptic mange [4]. Based on these findings, the purpose of the present study was to assess the efficacy of the topical administration of Certifect, against *Demodex canis* on dogs.

## Material and methods

The study was a single center trial including three parallel groups. The study design and the study conditions of this study were approved by the local animal welfare ethic committee in accordance with Good Clinical Practice by the European Agency for the Evaluation of Medicinal Products (CVMP/VICH GL9, July 2000; CVMP/VICH GL19, July 2001). All dogs belonged to private owners who signed an owner consent for the inclusion of their dogs in the study. Dogs were allocated to treatment groups randomly, and all evaluations of efficacy were performed by blinded personnel.

Eighteen mongrel dogs (12 males, 6 females) weighing from 6 to 19 kg and being at least 1-year old were included. All had clinical signs of *Demodex* infestation such as erythema, hair loss, seborrhea, follicular casts, scales, and crust in at least five spots or on an entire body region. Deep skin scrapings performed at the time of inclusion confirmed an average count of 527 *D. canis* mites for five lesion sites per dog. Morphological observations of the mites clearly identified them as *Demodex canis* type. Except for clinical signs of demodicosis, dogs were otherwise declared in good general condition by a veterinarian at the time of enrolment. The health condition of the dogs was monitored daily from inclusion (Day -7) to study end (Day 84). Significant secondary bacterial infections were reasons for exclusion.

Dogs were housed in individual cages preventing contact between animals and were exposed to ambient temperature and natural sunlight. Dogs had access to an individual outside run partly covered with a roof and were not exposed to rain. Food was provided in an amount and manner to maintain normal condition. Water was available *ad libitum*. Dogs were acclimatized to the study conditions for 7 days before the first treatment.

### Experimental design

- Dogs were randomized into three treatment groups based on decreasing mite counts at inclusion. Six dogs in treatment groups 1 were treated every four weeks with a topical formulation of Certifect (containing fipronil 6.26% w/v, amitraz 7.48% w/v, (S)-methoprene 5.63% w/v per complete volume of the dual cavity pipette) following the label recommendations (Table 1),

**Table 1.** Dosage recommendation for Certifect^®^ and Promeris Duo^®^.

Certifect^®^ dosage	
Bodyweight range (kg)	Pipette volumes (mL)
up to 10.0	1.07
10.1–20.0	2.14
20.1–40.0	4.28
Promeris Duo^®^ dosage	
Bodyweight range (kg)	Pipette volumes (mL)
5.1–10.0	1.33
10.1–25.0	3.33
25.1–40.0	5.33
- Six dogs in treatment group 2 were treated every two weeks with Certifect following the label-recommended dosage (Table 1),
- Six dogs in treatment group 3 were treated every four weeks with a topical formulation of Promeris Duo™ ^2^ (amitraz 15% w/v, metaflumizone 15% w/v) at the label-recommended dosage (Table 1).

Based on dog weight ranges, the maximum amitraz dosage for Promeris Duo is 50 mg/kg whereas it is 16 mg/kg for Certifect; the minimum dosages are 20 mg/kg for Promeris Duo and 8 mg/kg for Certifect.

Dogs in groups 1 and 3 were treated on days 0, 28, and 56 and dogs in group 2 on days 0, 14, 28, 42, 56, and 70.

### Mite counts

*Demodex* counts were performed on dogs prior to enrolment and on Days 27, 55, and 84. Each count consisted of a deep skin scraping from five lesion sites on each animal. Skin scraping sites were recorded (Table 2) and these sites were scraped at each subsequent examination. The surface of each skin scraping was 4 cm^2^ and they were made with a blade until capillary oozing occurred which took on average 3 min per site. The scrapings were mixed with oil and examined under a stereomicroscope. The number of live mites (adults and immatures) was counted. The methodology for mite count and clinical evaluation was similar to published efficacy data with other spot-ons in order to allow for comparison [2, 3].

**Table 2.** Localization of the five lesion sites retained for scraping sites on each dog.

DOG	Scraping site 1	Scraping site 2	Scraping site 3	Scraping site 4	Scraping site 5
1	Left shoulder	Right shoulder	Right side	Left side	Right buttock
2	Left shoulder	Right shoulder	Right side 1	Right side 2	Left side
3	Left shoulder	Right shoulder	Left side	Right side	Right buttock
4	Right shoulder	Right side	Left side	Left buttock	Right buttock
5	Left shoulder	Right shoulder	Left side	Right side	Right hind leg
6	Left shoulder	Right shoulder	Left side	Right side	Chest
7	Right shoulder	Right side	Left side	Left hind leg	Right hind leg
8	Left shoulder	Right shoulder	Left side	Right side	Right buttock
9	Right shoulder	Right side	Left side	Left buttock	Right buttock
10	Right shoulder	Right side	Left side	Left buttock	Right buttock
11	Right shoulder	Right side	Left side	Right buttock	Left buttock
12	Left shoulder	Right shoulder	Right side	Right buttock	Left buttock
13	Left shoulder	Right shoulder	Left side	Left buttock	Right buttock
14	Left shoulder	Right shoulder	Left side	Right side	Right buttock
15	Left shoulder	Right shoulder	Right side	Right buttock	Left buttock
16	Left shoulder	Right shoulder	Right side	Left side	Chest
17	Chest	Right side	Right buttock	Left side of chest	Left hind leg
18	Right shoulder	Right side	Left shoulder	Left side	Right hind leg

### Clinical evaluation

Clinical signs and the extent of demodectic lesions on each dog were assessed on the days when scrapings were made [2, 3]. Dogs were examined for the presence/absence of scales and crusts and the percentage of body area with hair regrowth. A semi-quantitative assessment of hair regrowth was made. The skin surface of the dogs on which hair regrowth occurred, compared to pre-treatment observations, was scored as 1 if < 50% hair regrowth occurred, 2 if 50–90% hair regrowth occurred, and 3 if > 90% hair regrowth took place.

### Data analysis

The primary efficacy variable was the decrease in live mite counts (adults and immatures) following treatment. The average percentage reduction in mite counts for the group was calculated by:

$$\mathrm{Reduction}\%\;\left(\mathrm{Group}\right)=(\mathrm{GM}\;\mathrm{inclusion}-\mathrm{GM}\;\mathrm{post}-\mathrm{treatment})/\mathrm{GM}\;\mathrm{inclusion}\times 100$$

where GM Inclusion = the geometric mean (GM) of the Inclusion (pre-treatment) mite counts, and GM Post-treatment = the geometric mean of the Post-treatment mite counts.

The Inclusion and Post-treatment mite counts within each group were compared using an ANOVA with time (pre- or post-treatment) and dog effects. A 5% level of significance for the within-group comparison (pre-treatment live mite counts vs post-treatment live mite counts) was used. The three groups were compared descriptively with respect to the percent reduction in mite counts. The success rate was defined as the percentage of dogs in each group that were negative for live mites at the time of scraping.

The secondary variable was the resolution of clinical signs. The number of dogs in each group affected by scales and crusts was tabulated at inclusion and at the different post-treatment assessment days. In addition, the number of dogs in each category of hair regrowth was tabulated by group for each post-treatment evaluation.

## Results

### Antiparasitic efficacy

All the dogs were positive for the presence of live mites prior to the first treatment. For all three treated groups on all post-treatment assessment days, the mite counts were statistically significantly reduced (*p* < 0.05) compared to the counts at inclusion (Table 3). At the first post-treatment evaluation (Day 27), the percentage reduction in the three groups ranged from 97.4 to 97.9%, with two dogs with no mites identified in groups 1 and 2 each.

**Table 3.** Individual *Demodex* counts for each dog at each evaluation and mean percentage of mite count reduction for each group.

		Day -2	Day 27	Day 55	Day 84
Group 1: combination of fipronil, amitraz, and (S)-methoprene at 28-day intervals	Dog 1*	2,454	-	-	-
	Dog 2	796	0	10	2
	Dog 3	443	115	191	2
	Dog 4	238	15	0	0
	Dog 5	158	12	0	0
	Dog 6	68	0	0	0
	Geometric mean	361.7	6.5	3.6	0.6
	Percent reduction		97.4%	98.5%	99.8%
Group 2: combination of fipronil, amitraz, and (S)-methoprene at 14-day intervals	Dog 7	1,335	7	3	0
	Dog 8	722	129	1	0
	Dog 9	504	69	0	0
	Dog 10	368	0	0	0
	Dog 11	77	0	0	0
	Dog 12	20	1	0	0
	Geometric mean	257.0	6.3	0.4	0.0
	Percent reduction		97.6%	99.8%	100.0%
Group 3: combination of amitraz and metaflumizone at 28-day intervals	Dog 13	842	11	0	0
	Dog 14	615	2	1	0
	Dog 15	436	16	0	0
	Dog 16	327	1	0	0
	Dog 17	68	5	0	0
	Dog 18	9	1	0	0
	Geometric mean	191.8	3.9	0.1	0.0
	Percent reduction		97.9%	99.9%	100.0%

The post-treatment number counts differed significantly (*p* < 0.05) from the Inclusion mean mite counts on all post-treatment assessment days for all three treated groups.

* Dog 1 died during the second week (Day 12) from its generalized demodicosis.

On Day 55, most of the dogs did not harbor *Demodex* mites and the percentage reductions were 98.5, 99.8, and 99.9% in groups 1, 2, and 3, respectively.

On Day 84, none of the dogs in groups 2 and 3 harbored *D. canis*, resulting in 100% efficacy. The percentage reduction in group 1 was 99.8% as two dogs still had two mites each.

The difference in mite counts between the three groups was not statistically significant at any time point. Three, four, and five dogs in treatment groups 1, 2, and 3, respectively, had two consecutive negative scrapings at the one-month interval which is considered a cure by dermatologists.

### Clinical efficacy

Clinical signs of demodicosis were markedly reduced following the three treatment regimens and correlated with the mite count reductions (Table 2). Scales and crusts completely disappeared in 5/5 dogs in group 1, 3/6 dogs in group 2, and 5/6 dogs in group 3.

The sizes of the affected areas were considerably reduced as shown by hair regrowth (Table 4). On Day 84, all dogs in groups 1 and 2 had a body area with hair regrowth over 90% compared to that recorded during inclusion.

**Table 4.** Evaluation of the clinical signs associated with demodicosis and hair regrowth during the 3-month study period.

	Day -2	Day 27				Day 55				Day 84			
	Number of dogs with crusts or scales/total	Number of dogs with crusts or scales/total	Hair regrowth score* (number of dogs in each category)			Number of dogs with crusts or scales/total dogs	Hair regrowth score (number of dogs in each category)			Number of dogs with crusts or scales	Hair regrowth score (number of dogs in each category)		
			1	2	3		1	2	3		1	2	3
Group 1: combination of fipronil, amitraz, and (S)-methoprene (28 Days)	6/6	3/5*	4	1	0	2/5	0	5	0	0/5	0	0	5/5
Group 2: combination of fipronil, amitraz, and (S)-methoprene (14 Days)	6/6	4/6	3	3	0	6/6	0	5	1	3/6	0	0	6/6
Group 3: combination of amitraz and metaflumizone (28 Days)	5/6	4/6	3	1	2	2/6	0	3	3	1/6	0	1	5/6

Hair regrowth score = The skin surface of the dogs on which hair regrowth occurred, compared to pre-treatment observations, was scored as 1 if <50% hair regrowth occurred, 2 if 50–90% hair regrowth occurred, and 3 if >90% hair regrowth took place.

* One of the group 1 dogs died at Day 12 for a reason not related to treatment.

### Health observations

No adverse events related to treatments were observed. One dog (4C5 C66) in study Group 1 was found dead in its cage on Day 12 and the cause was determined from post mortem examinations as being the consequences of demodicosis with secondary bacterial infection and septicaemia.

## Discussion and conclusion

The present study demonstrates that treatment with a spot-on formulation of fipronil, amitraz, and (S)-methoprene (Certifect) for 3 months at bi-weekly or monthly intervals resulted in a rapid reduction in mite numbers and a marked improvement in clinical signs in all dogs. A significant portion of group 1 (3/5, 66.7%) and group 2 (6/6, 100%) had no mites in their skin scrapings at the end of the study. The sensitivity of skin scrapings to detect remission of demodicosis has sometimes been challenged. As the life cycle of the mite extends over a period of 18–24 days [16], and considering that scrapings are performed on a limited area of the lesions, a single negative skin scraping should generally not be considered as an indication of complete remission. Remission should rather be determined based on two consecutive negative skin scrapings done at a one-month interval [5, 9]. In the present study, the majority of dogs had two negative skin scrapings, indicating that treatments at appropriate intervals can provide remission of disease. Unfortunately, the duration of the study did not allow a second evaluation of the three dogs that had a few mites at Day 55 and no mites at Day 84. The clear clinical improvement seen on all dogs is another sign of effective treatment. It is known that even without mites, the lesions will disappear slowly in some dogs due to the time needed for skin to fully recover.

Both monthly and bi-weekly treatments with Certifect were regarded as effective in treating dogs with generalized demodicosis. Bi-weekly treatments did however result in a complete disappearance of mites in the scrapings at Day 84.

This study demonstrates that both monthly and bi-weekly applications of Certifect over a period of 70 days are effective in treating dogs with generalized demodicosis. *Demodex* mite infestations were quickly reduced, resulting in a marked clinical improvement. These results are similar to other published data assessing the efficacy of a metaflumizone-amitraz combination [3]. In this study, the effectiveness of Certifect treatments was comparable to that of Promeris Duo. The Certifect treatment applies an amitraz concentration on dogs that is in average 40% of the Promeris Duo treatment. The comparable effect is probably due to a potentiation with fipronil.

## Competing interests

This clinical study was funded by Merial S.A.S., 29 avenue Tony Garnier, 69007 Lyon of which Frederic Beugnet, Lénaïg Halos and Matthias Pollmeier are employees. ClinVet, of which J.J. Fourie is an employee, is an independent, South African, Contract Research Organisation contracted to conduct the study. All authors voluntarily publish this article and have no personal interest in these studies other than publishing the scientific findings that they have been involved in via planning, initiating, monitoring, and conducting the investigations and analyzing the results.
